# Identification of novel ΔNp63α-regulated miRNAs using an optimized small RNA-Seq analysis pipeline

**DOI:** 10.1038/s41598-018-28168-5

**Published:** 2018-07-03

**Authors:** Suraj Sakaram, Michael P. Craig, Natasha T. Hill, Amjad Aljagthmi, Christian Garrido, Oleg Paliy, Michael Bottomley, Michael Raymer, Madhavi P. Kadakia

**Affiliations:** 1 Biochemistry and Molecular Biology, Wright State University, Dayton, OH 45435 USA; 2 Math and Microbiology, Wright State University, Dayton, OH 45435 USA; 3 Computer Science and Engineering, Wright State University, Dayton, OH 45435 USA

## Abstract

Advances in high-throughput sequencing have enabled profiling of microRNAs (miRNAs), however, a consensus pipeline for sequencing of small RNAs has not been established. We built and optimized an analysis pipeline using Partek Flow, circumventing the need for analyzing data via scripting languages. Our analysis assessed the effect of alignment reference, normalization method, and statistical model choice on biological data. The pipeline was evaluated using sequencing data from HaCaT cells transfected with either a non-silencing control or siRNA against ΔNp63α, a p53 family member protein which is highly expressed in non-melanoma skin cancer and shown to regulate a number of miRNAs. We posit that 1) alignment and quantification to the miRBase reference provides the most robust quantitation of miRNAs, 2) normalizing sample reads via Trimmed Mean of M-values is the most robust method for accurate downstream analyses, and 3) use of the lognormal with shrinkage statistical model effectively identifies differentially expressed miRNAs. Using our pipeline, we identified previously unrecognized regulation of miRs-149-5p, 18a-5p, 19b-1-5p, 20a-5p, 590-5p, 744-5p and 93-5p by ΔNp63α. Regulation of these miRNAs was validated by RT-qPCR, substantiating our small RNA-Seq pipeline. Further analysis of these miRNAs may provide insight into ΔNp63α’s role in cancer progression. By defining the optimal alignment reference, normalization method, and statistical model for analysis of miRNA sequencing data, we have established an analysis pipeline that may be carried out in Partek Flow or at the command line. In this manner, our pipeline circumvents some of the major hurdles encountered during small RNA-Seq analysis.

## Introduction

MiRNAs are small non-coding RNAs of approximately 18–22 nucleotides in length that bind to the 3′ UTR regions of target mRNA to translationally repress or degrade them^1^. A single miRNA is capable of targeting hundreds of genes and it is estimated that they may regulate over a third of all mammalian genes. Thus, the dysregulation of several miRNAs can have strong biological effects on entire gene networks^1,2^. MiRNAs regulate a number of cellular processes that are dysregulated in cancer, such as proliferation, differentiation, apoptosis, motility and invasion. Further, changes in miRNA expression profiles reflect the developmental lineage and differentiation state of cancers, and are thus being used as cancer biomarkers^3,4^. In recent years, improvements in Next Generation Sequencing (NGS) have made it possible to sequence small RNA species like miRNA with unprecedented sensitivity and dynamic range. Despite the increasing use of small RNA-Sequencing (small RNA-Seq) to identify potential biomarkers and therapeutic targets for cancer, there is no consensus on a data analysis pipeline for miRNA-Seq data^5–7^.

Standard NGS data analysis assumptions and algorithms used in mRNA sequencing experiments are routinely used in small RNA-Seq experiments despite inherent differences in read length, read depth and coverage between mRNA and miRNA datasets^8^. For example, seed lengths greater than 25 nucleotides are commonly used in RNA-Seq analyses, however, these seed lengths are longer than the average miRNA length and are not appropriate for aligning miRNA. Alignment of small RNA-Seq data requires the use of shorter seed lengths. Consequently, this increases the likelihood of individual reads mapping to multiple locations thereby increasing the uncertainty in mapping and quantitating reads. Further, given the uniform length of miRNAs, it is possible that normalization methods such as RPKM, which correct for differences in read length, may negatively impact analysis of miRNA datasets. Despite these limitations, the choice of alignment index, quantitation reference, and normalization method to identify differentially expressed miRNAs from small RNA-Seq data have not been fully evaluated. Validating a standard pipeline for small RNA-Seq data is critical since each step of processing impacts downstream analysis and identification of statistically significant differentially expressed (DE) miRNAs.

In this study, small RNA-Seq was used to identify novel ΔNp63α-regulated miRNAs in keratinocytes by comparing those in which ΔNp63α was silenced relative to non-silencing controls (NSC). ΔNp63α, a member of the p53 family, has been shown to modulate the expression of miRNAs involved in various cellular processes including the regulation of keratinocyte differentiation, cell migration, tumor growth, cell cycle arrest, apoptosis, and metabolism^9–11^. ΔNp63α regulates miRNAs by directly regulating their transcription, and indirectly through the regulation of other transcription factors, such as the Runt-related transcription factor 1 (RUNX1), which in turn regulates miRNAs^12,13^. Importantly, ΔNp63α has also been shown to regulate the global post-transcriptional processing of miRNAs by transcriptionally activating DGCR8 in the Drosha complex and potentially through direct interaction with the Drosha PY-WW domain^14^. Thus, the dysregulation of ΔNp63α in epithelial cancers alters miRNA expression through a variety of indirect and direct mechanisms, and it is our hope that ΔNp63α-regulated miRNAs may serve as novel cancer biomarkers or therapeutic targets.

To identify miRNAs that are differentially expressed between keratinocytes lacking ΔNp63α versus expressing ΔNp63α, we optimized key parameters in the analysis to develop a standard pipeline for analyzing small RNA-Seq data. In this study, we identified several novel miRNAs regulated by ΔNp63α using our optimized pipeline. A sub group of these miRNAs were validated by RT-qPCR further supporting the pipeline we established. Analysis of the functional roles of these miRNAs and their targets will facilitate a deeper understanding of ΔNp63α’s role in both maintaining epidermal integrity and determining tumorigenic fate. Our results will provide non-bioinformaticians, who rely on sequencing analysis software for their research, an optimized small RNA-Seq pipeline to expedite data analysis, thereby enabling researchers to focus on the biological significance of their findings. Furthermore, the pipeline parameters chosen herein (e.g. alignment and quantitation to the miRBase reference, TMM normalization and use of an LNS model for identification of differentially expressed miRNAs) may be implemented at the command line with the use of open-source tools for small RNA-Seq analysis.

## Results

### Small RNA Sequencing for miRNAs in HaCaT cells with p63 knockdown

**Figure 1:**
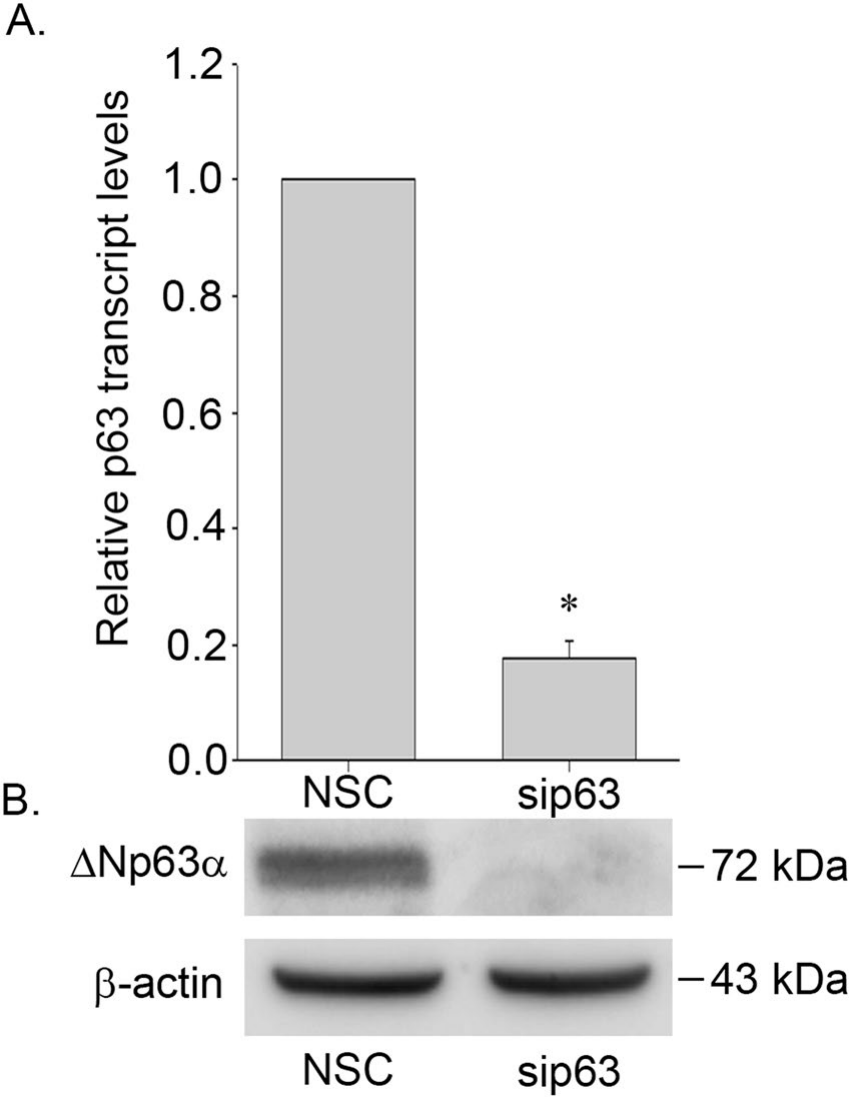
ΔNp63α was silenced in HaCaT samples used for small RNA-Seq. HaCaT cells were transfected with either non-silencing control siRNA or siRNA against p63. (**A**) RT-qPCR analysis of p63 transcript levels normalized to endogenous GAPDH. (**B**) p63 protein levels in NSC and sip63 cell lysates. β-actin was included as a loading control to show an equivalent amount of protein was added in each lane. MW in kDa is indicated to the right of each blot. Testing was performed in triplicate (n = 3) for each of the 3 biological replicates. Error bars indicate +1 SD. Asterisk indicates p ≤ 0.05 by Student’s T-test.

To examine miRNAs regulated by ΔNp63α, we transfected HaCaT cells with either non-silencing control siRNA (NSC) or p63 specific siRNA. These cells express ΔNp63α, the most physiologically relevant isoform of p63 expressed in the basal layer of the skin^15–17^. All three biological replicates of HaCaT cells transfected with siRNA against p63 (sip63) showed 80% or greater reduction in p63 transcript levels by RT-qPCR (Fig. 1A) and no detectable p63 protein by immunoblot (Fig. 1B) relative to non-silencing control (NSC) (representative data shown), thus confirming p63 knockdown. Bioanalyzer measurements showed that our samples had an average of 7–11% RNA of 10–40 nucleotides in length, which we considered to be miRNAs per the manufacturer’s guideline. After size-selection and library preparation, barcoded cDNA libraries prepared from each of the 3 biological replicates of HaCaT cells transfected with NSC and sip63 were pooled and sequenced yielding over 6 million reads per sample (data not shown). Mean read lengths between 20 and 25 base pairs were obtained, consistent with expected miRNA length.

### Analysis of small RNA-Seq data

**Figure 2:**
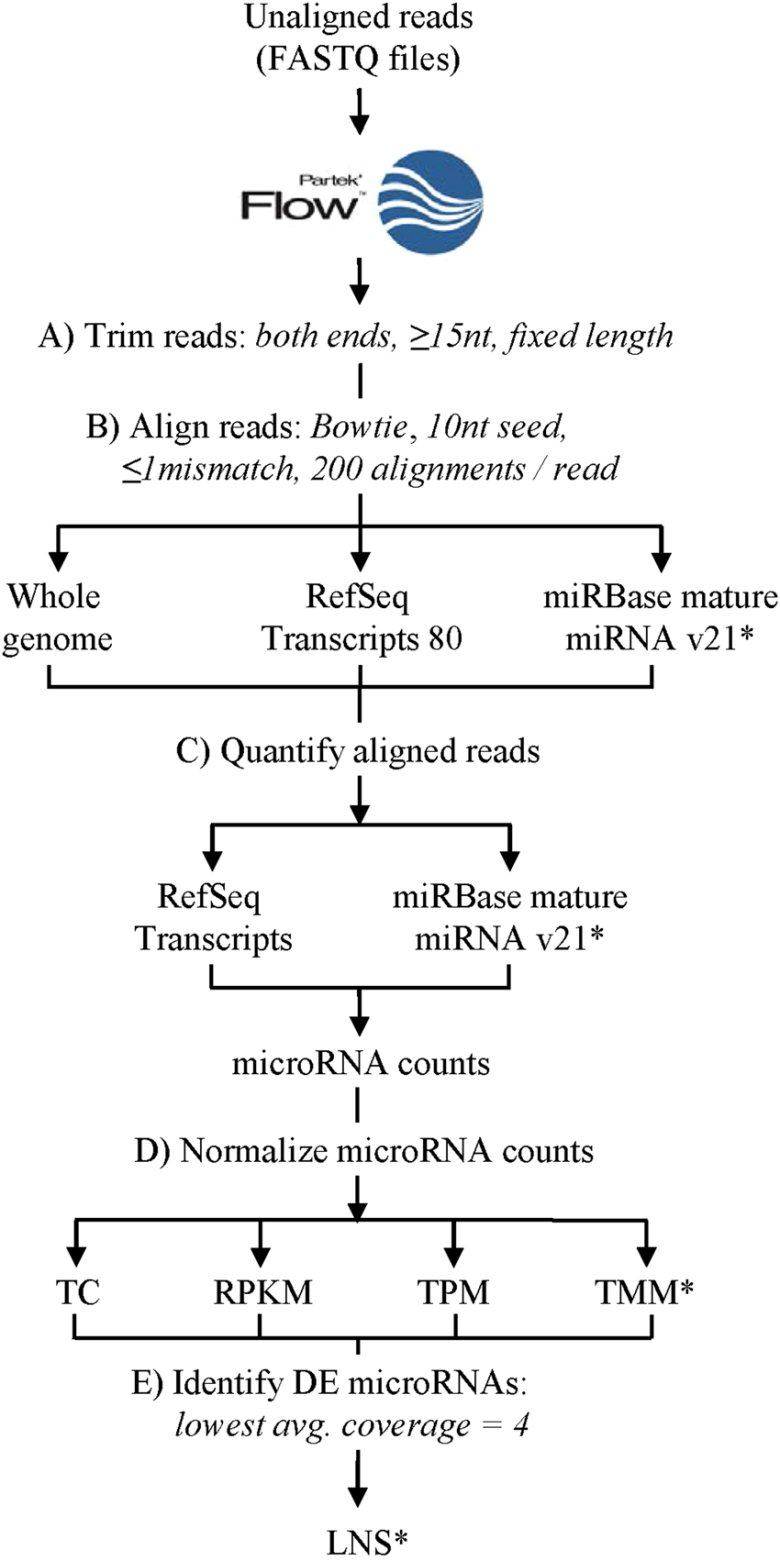
Data processing workflow. Schematic of workflow for evaluating different combinations of alignment references and normalization procedures considered in our studies. Raw FASTQ files were processed using Partek Flow by (**A**) trimming reads to a fixed length based on PHRED quality score, (**B**) aligning reads and (**C**) quantitating these reads to one of the reference databases listed, (**D**) normalizing read counts, and (**E**) identifying differentially expressed miRNA. Asterisks indicate components of the optimized pipeline.

The general workflow for small RNA-Seq analysis used in this study, including alignment, quantitation, normalization, and differential gene expression analysis is shown in Fig. 2. The choice of alignment index, quantitation reference, normalization method, and statistical probability distribution model are known to affect differential expression analyses of miRNA datasets, thus highlighting the need for careful consideration of pipeline parameters during small RNA-Seq analysis^8,18,19^. Figure 2 shows the key steps in the data analysis workflow evaluated during small RNA-Seq pipeline optimization.

To approximately determine the total miRNA content in each sample, trimmed reads were first aligned and quantitated to the miRBase reference to obtain the total miRNA read count for each sample. Reads that did not align using the miRBase reference were re-aligned to the whole genome reference and quantitated using the RefSeq reference. Re-aligning to a different reference in this manner served to identify as many potential miRNAs as possible and aided in the quantitation of other RNA species. In combination with the miRNA reads quantitated on the first pass, we estimate that roughly 71 ± 6% of reads were miRNAs and 9 ± 4% were snoRNAs, with the remainder likely comprised of degradation products and other low-quality reads.

To determine the optimal alignment index and quantitation reference, we assessed the differences in total aligned reads, quantitated reads, and the number of unique miRNAs in each of the 6 combinations tested as shown in Table 1. As expected, the total number of aligned reads was greatest when aligned to the whole genome (hg38) and lowest when aligned to the miRBase reference, reflecting the number of annotations present in each reference. For reads aligned to either whole genome or RefSeq, the number of quantitated reads was higher when using RefSeq as the quantitation reference. This is attributed to the fact that size selection for small RNA during our library preparation retained other types of small RNAs (e.g. piRNA, snoRNA, etc.) as well as partially degraded mRNA. As expected, the number of aligned reads and quantitated reads were identical when miRBase was used as the alignment reference, independent of quantitation reference. Despite differences in the total number of aligned reads between pipelines, all pipelines identified a large number of unique miRNA, a more direct indication of pipeline performance. Interestingly, in all 6 conditions tested, the number of unique miRNAs quantitated was the highest when miRBase was used as the alignment and quantitation reference.

**Table 1:** Effect of alignment reference on read quantification.

Alignment reference	Condition	Total aligned reads (x 1000)	Quantified reads (x1000)	# of Unique miRNA quantitated
**a**. **RefSeq quantitation**				
Whole Genome	NSC	3,482 ± 434	2,735 ± 320	1776
	sip63	2,979 ± 487	2,328 ± 406	1816
RefSeq	NSC	2,681 ± 292	2,681 ± 292	1874
	sip63	2,295 ± 398	2,295 ± 398	1873
miRBase	NSC	676 ± 113	676 ± 116	1802
	sip63	526 ± 138	526 ± 138	1806
**b**. **miRBase quantitation**				
Whole Genome	NSC	3,482 ± 434	1,039 ± 59	1327
	sip63	2,979 ± 487	661 ± 124	1309
RefSeq	NSC	2,681 ± 292	1,080 ± 59	2241
	sip63	2,295 ± 398	705 ± 130	2276
miRBase	NSC	676 ± 116	676 ± 116	2582
	sip63	526 ± 138	526 ± 138	2586

Raw reads from NSC or sip63 samples trimmed to a fixed length based on quality score were aligned to the whole genome, RefSeq, or miRBase references and quantitated using either (a) RefSeq or (b) miRBase reference. The total number of aligned reads indicate the total number available for quantitation. The number of quantified and unique reads obtained using each of these methods is also indicated.

**Figure 3:**
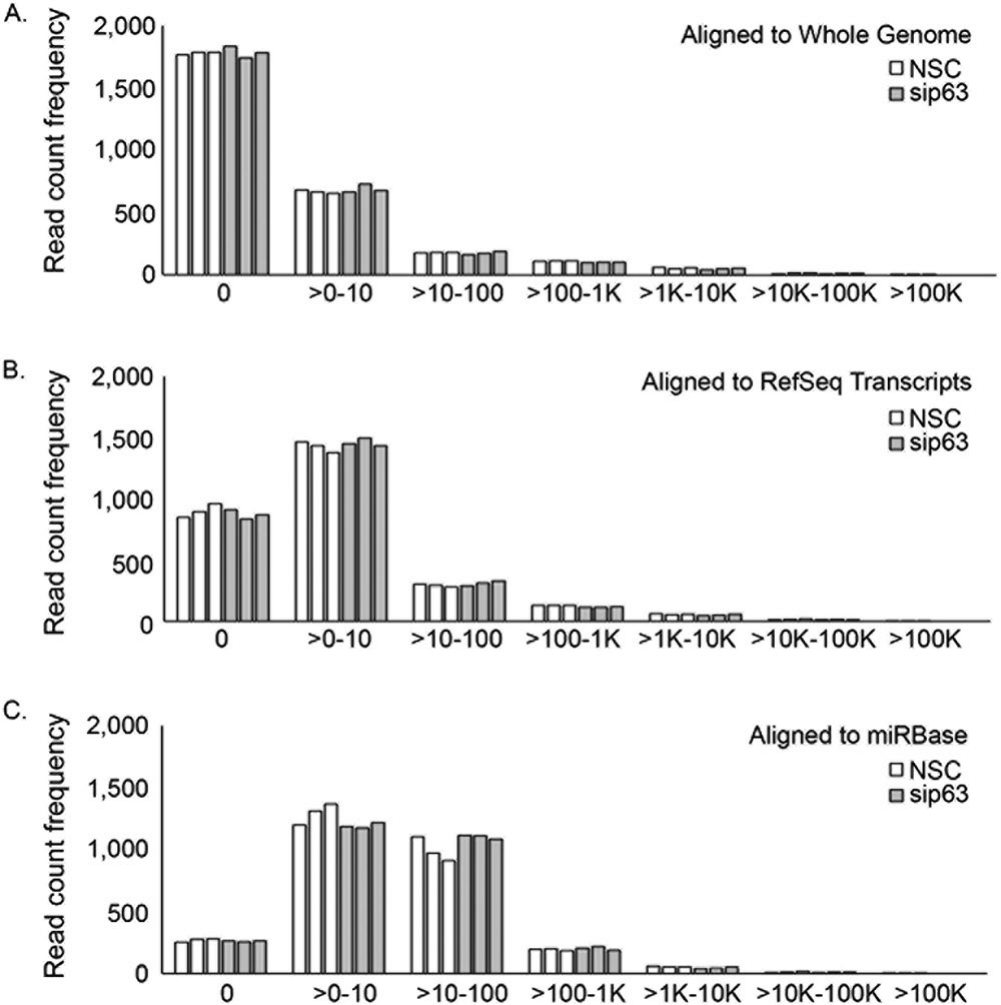
Alignment and quantitation to miRBase provides the most robust read quantitation. The frequency of raw read counts from each of the 3 biological replicates for the NSC and sip63 datasets is shown for (**A**) Whole Genome, (**B**) RefSeq and (**C**) miRBase alignments after quantitation using the miRBase reference. Open and gray bars indicate NSC and sip63 samples, respectively.

**Figure 4:**
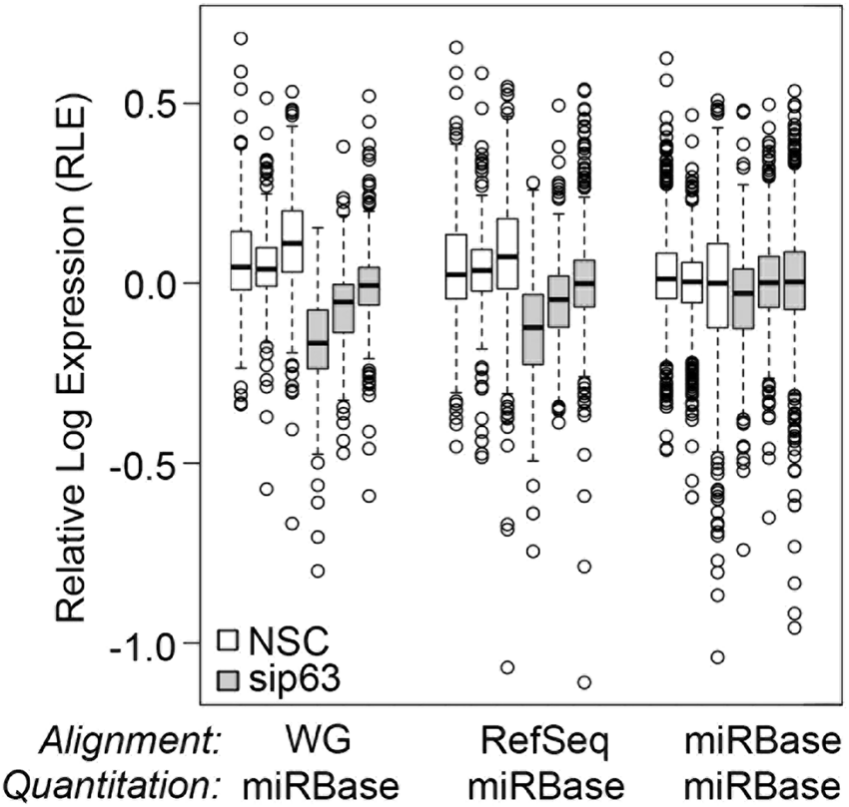
Alignment using miRBase is optimal for small RNA-Seq analysis of miRNAs. Raw read counts filtered to remove miRNA with <10 reads in any individual NSC or sip63 sample were used to calculate relative log expression (RLE) values as indicated in materials and method section. Box plots of relative log expression (RLE) values for each sample are shown on the y-axis with median, quartiles, +/−1.5 interquartile range, and outliers indicated by the middle line, box border, whiskers, and circles, respectively. Open and gray bars indicate NSC and sip63 samples, respectively.

To further assess the difference between alignment indexes, we looked at the frequency of raw read count values for each index when quantitated using miRBase (Fig. 3). When aligned to Whole genome and RefSeq, a significant number of miRNAs received 0 counts, indicating that approximately 63% and 30% of miRNAs contained in the miRBase reference, respectively, were not quantitated (Fig. 3A,B). By contrast, when aligned to miRBase, a majority of the miRNAs (~88%) received counts between 10 and 1,000 reads (Fig. 3C). Thus, more miRNAs were quantitated using miRBase as the alignment index. To further compare the distribution of raw read counts, box plots were generated from Relative Log Expression (RLE) values calculated for each of the miRBase quantitated datasets. Assuming that gross variation in raw read count distributions is primarily due to differences in library preparation and sequencing efficiency, one would expect sample replicates to have similar median values and roughly similar distributions of raw read counts such that the median RLE values are distributed around zero^20^. While reads aligned to whole genome or RefSeq show more variation in median RLE values, the miRBase aligned reads show a median RLE which was naturally centered at zero and a uniform distribution across samples (Fig. 4). Together, these results clearly demonstrate that miRBase is the optimal alignment index and quantitation reference.

**Figure 5:**
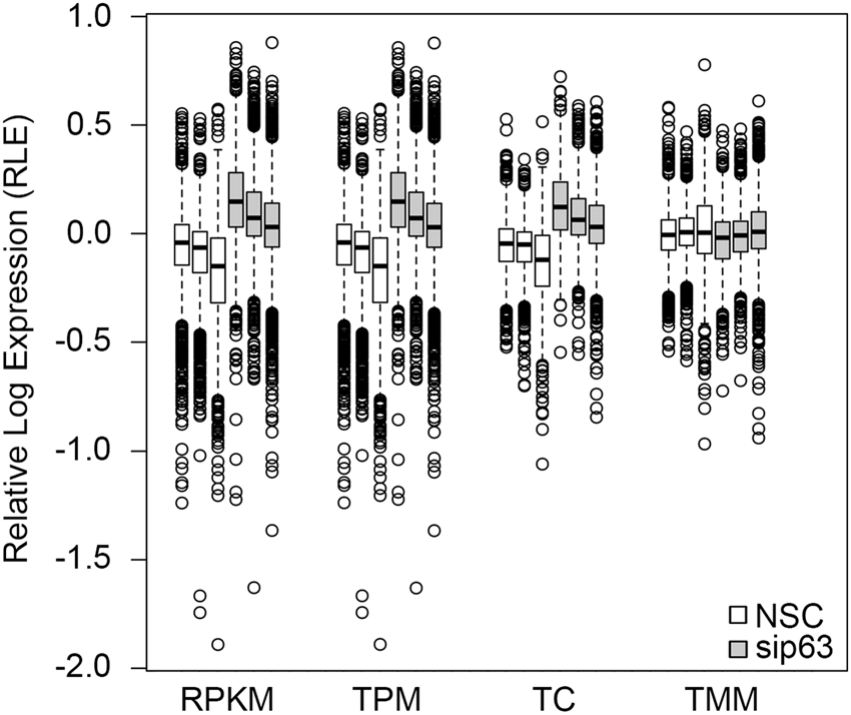
TMM is the most effective normalization for data aligned and quantitated to miRBase. Normalized read counts obtained following normalization to RPKM, TPM, TC and TMM were used to calculate RLE values as indicated in materials and method section. Box plots of relative log expression (RLE) values for each sample are shown on the y-axis with median, quartiles, +/−1.5 IQR, and outliers indicated by the middle line, box border, whiskers, and circles, respectively. Open and gray bars indicate NSC and sip63 samples, respectively.

Next, to determine the optimal normalization method for our miRNA datasets, miRNA read counts were normalized using each of four normalization methods: RPKM, TPM, TC, and TMM. To compare the effect of each normalization algorithm, box plots were generated from RLE values of normalized reads (Fig. 5). One would expect that effective normalization would produce similar median values within treatment groups and roughly similar distributions of raw read counts for all samples^20^. Normalization of miRBase aligned and quantitated data using RPKM or TPM showed increased variation in the median RLE compared to TC or TMM normalized data. Normalization by TC reduced variance but failed to stabilize the median RLE values across samples. TMM outperformed the other normalization methods by reducing variance and stabilizing the median RLE values around zero (Fig. 5).

**Figure 6:**
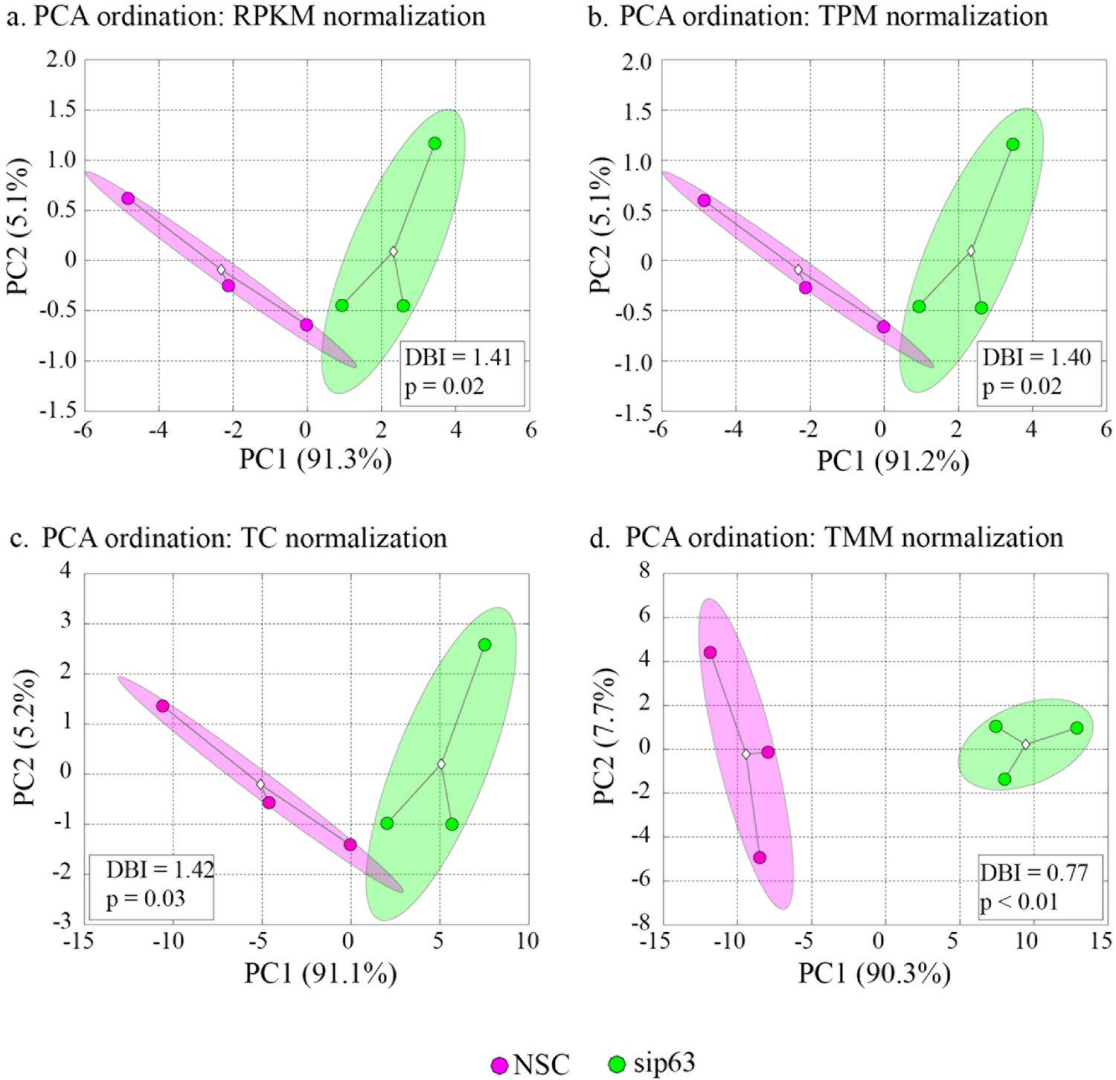
TMM normalization yields the best clustering of NSC and sip63 samples. Panels a through d show the assessment of sample similarity by principal components analysis (PCA) of the normalized miRNA datasets. Different groups are denoted by colors as shown in the legend. Group clouds represent areas of three standard errors around the group centroid (diamond). Percent of total variance captured by each principal component is shown in parentheses. Statistical significance of group separation in PCA space was assessed as described in materials and methods.

Finally, Principal Components Analysis (PCA) was performed to assess the similarity of miRNA expression profiles among samples. Figure 6 displays the PC1-vs-PC2 scatter plots of PCA output for each tested normalization procedure. In all cases, sip63 and NSC groups separated along the PC1 axis, indicating that the differences between groups accounted for the largest observed variance in the dataset. Variance among samples within each group was distributed along the PC2 axis. While the dispersion of samples and corresponding Davies-Bouldin (DB) index measures were comparable for RPKM, TPM, and TC methods, TMM normalization led to an improved separation of sip63 and NSC samples in PCA space (as evidenced by the lower DB index and p value, see Fig. 6). Due to the median values being most closely centered around zero, from the data presented by the PCA analyses, TMM normalization method appears to be superior.

Altogether, thus far, we have demonstrated that alignment and quantitation of trimmed reads using miRBase results in mapping the greatest number of unique miRNAs. We further showed that normalizing raw reads via TMM is optimal for downstream analyses. To identify differentially expressed miRNAs by ΔNp63α, we focused on the normalized reads obtained from this pipeline configuration.

### Identification of Differentially Expressed miRNAs

**Figure 7:**
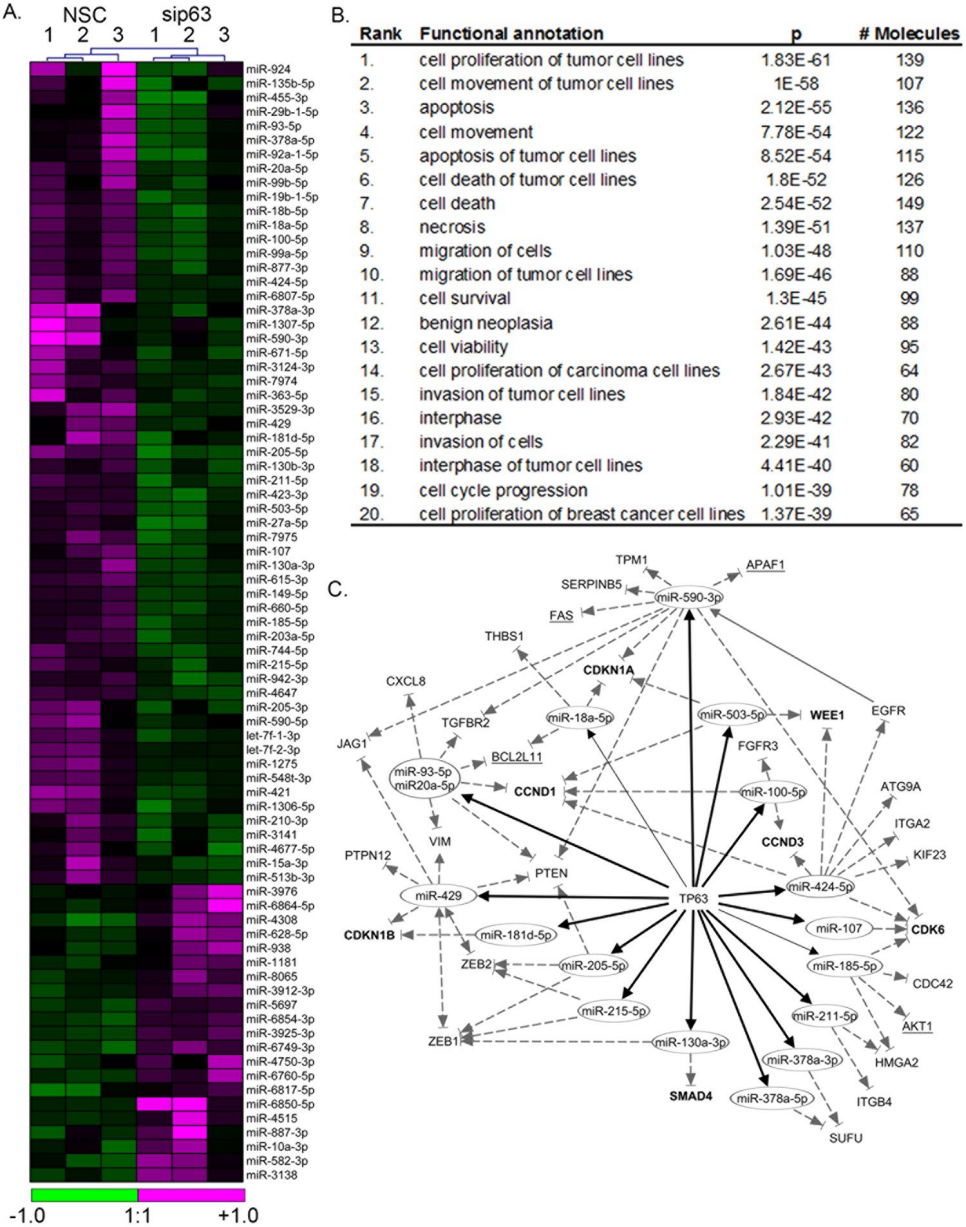
Heat map and functional analysis of miRNA differentially expressed in response to ΔNp63α knockdown. (**A**) Heat map of the 79 total miRNAs identified as differentially expressed (reads ≥10, p ≤ 0.05, fold change ≥1.5) generated in Partek Flow using miRBase aligned and quantitated, TMM normalized data modeled using LNS. (**B**) Functional profiling of the 245 predicted mRNA targets of these ΔNp63α -regulated miRNA performed in Ingenuity Pathway Analysis using their database of experimentally validated target mRNA. (**C**) Signaling network showing ΔNp63α -regulated miRNA and their respective mRNA targets identified using the IPA knowledge base of experimentally validated targets. The network is filtered to include only mRNA targets known to be downstream of ΔNp63α. Bold lines indicate novel miRNA positively regulated by p63 which were identified by NGS; regular (not bold) lines indicate known miRNA positively regulated by p63. Dotted lines with arrowheads and flat endcaps indicate mRNA targeted by miRNA (either direct or indirect). The 4 mRNA targets involved in apoptosis are underlined, and the 7 involved in cell cycle are in bold.

Differential expression (DE) analysis of the miRNAs was performed between the NSC and sip63 samples using the LNS model. Selection criteria used to identify these miRNAs were reads ≥10 in each sample, FC ≥1.5, and p ≤ 0.05. The full list of 79 miRNA that are predicted to be positively and negatively regulated by ΔNp63α are provided in Supplementary Tables 1 and 2, respectively. These tables show the average read counts for NSC and sip63 triplicates, p-values calculated using the LNS model and fold change. Of the 79 miRNAs identified, 58 miRNAs were positively regulated whereas 21 miRNAs were negatively regulated by ΔNp63α. Figure 7A shows a heat map of the differences in expression level for these 79 differentially expressed miRNAs in all 6 samples after both samples and features were clustered using a Euclidean distance metric and average-linkage algorithm. NSC and sip63 samples clustered together with up- or down-regulation of a majority of miRNA correlating with sample type. This list of miRNA was compared to a previously published dataset of p63-regulated miRNAs summarized in our previous study^21^. Eight of the currently identified miRNA were previously shown to be regulated by ΔNp63α and served as positive controls (miR-185–5p, miR-205-5p, miR-130b-3p, miR-203a-5p, and miR-429)^10,22–24^. Additionally, seven ΔNp63α-regulated miRNA have been specifically identified in HaCaT cells: miR-17, miR-18a, miR-20b, miR-30a, miR-106a, miR-143, miR-455-3p^25–28^. Of these, only miR-455-3p and miR-18a-5p met the reads, fold change and p-value cutoff used in the chosen pipeline. miR-20b was detected at very low levels in our samples and did not meet our strict filtering criteria. Consistent with previous reports, miR-17, miR-30a, miR-106a and miR-143 were identified as being positively regulated by p63 but did not reach statistical significance in our dataset and therefore excluded from our final list (Supplementary Table 1).

### Pathway analysis of differentially expressed miRNAs

miRNAs shown to be regulated by ΔNp63α (Supplementary Tables 1 and 2) were subjected to pathway analysis. mRNA targets for these 79 miRNAs were identified in Ingenuity Pathway Analysis using a database of experimentally validated targets. A functional analysis of these targets indicated significant enrichment of genes involved in cell proliferation, movement, and apoptosis (p < 0.001) (Fig. 7B). These cellular functions have previously been identified as being affected by silencing of ΔNp63α and are consistent with the known functional role of ΔNp63α in maintaining epithelial stemness and promoting cancer progression.

Using IPA Upstream Regulator analysis tools, p63 (TP63) was identified as a significant upstream regulator of the experimentally validated targets of the 79 differentially expressed miRNA (p < 0.001). Thirty of these mRNA targets were previously known targets of p63 listed in the IPA Knowledge Base. These mRNA were linked to the 16 corresponding p63-regulated miRNA known to target them in the p63 signaling network shown in Fig. 7C. Among the 30 mRNAs predicted to be downstream of p63, 4 are involved in apoptosis and 7 in cell cycle (Fig. 7C).

### Validation of statistically significant DE miRNAs

**Figure 8:**
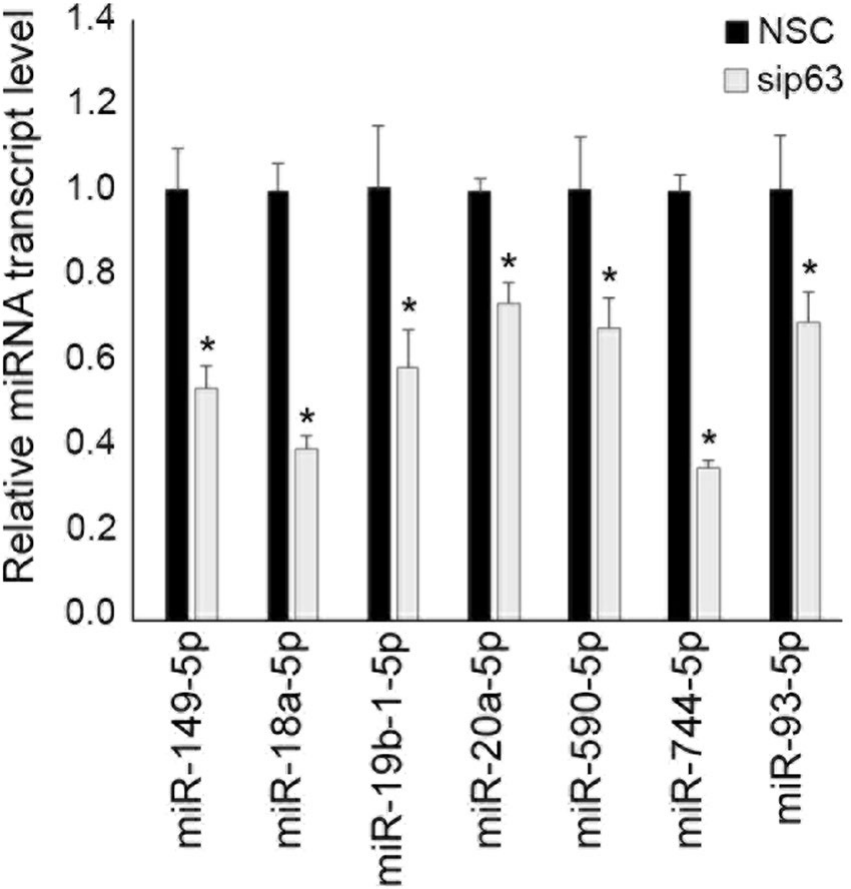
Validation of candidate miRNAs identified by sequencing using RT-qPCR. RT-qPCR was performed in triplicate for each specific miRNA for each of the 3 biological replicates of NSC and sip63 transfected HaCaT cells. Expression levels were normalized to RNU48 and shown relative to NSC. Error bars indicate +1 SD. Asterisks indicate p < 0.05 calculated by Student’s T-test.

From the list of 79 miRNAs with significant changes in expression (p ≤ 0.05, reads ≥10 for all samples), those with reads greater than 250 in each of the NSC samples were subjected to validation to assess the robustness of the established pipeline. Out of these, a sub-group of miRNAs not shown to be previously regulated by p63 were shortlisted for validation for novel discovery. miR-149-5p, 18a-5p, 19b-1-5p, 20a-5p, 590-5p, 744-5p and 93-5p were validated by RT-qPCR. RT-qPCR results revealed that all 7 miRNAs were downregulated when ΔNp63α was silenced, thus confirming the DE results predicted by small RNA-Seq that these miRNAs are positively regulated by ΔNp63α (Fig. 8).

### Testing of small RNA-Seq pipeline using a publicly available dataset

A previous study by Zhao *et al*.^29^ performed on the Illumina platform identified 173 miRNA which were differentially expressed between early and mid-gestational fetal keratinocytes. Moreover, the authors validated a total of 10 by RT-PCR. While 4/10 are known miRNAs, 6 represent novel miRNA not listed in miRBase v18. Reanalysis of the FASTQ files obtained from the same study (SRA GSE65342) was performed using our optimized pipeline parameters except for using hg19 and miRBase v18 references to be consistent with that study. Our analysis led to the identification of 161 differentially expressed miRNA, 79 of which were also identified by Zhao *et al*. and includes the 4 known microRNA validated by PCR in that study. Of the remaining 94/173 not detected in our analysis, 52 of them had low read counts (<10 in all samples) and would not have been selected for analysis based on our stringent filtering criteria. Although there is a large concordance in the results obtained following analysis by both pipelines, it is not uncommon that we see unique microRNAs picked up by different analysis methods. This analysis provides additional empirical data in support of the current pipeline design and function with biological data collected using a different sequencing platform.

## Discussion

In this study, we sought to optimize a user-friendly pipeline for small RNA-Seq analysis which could be utilized with little to no command line experience. We used HaCaT cells where ΔNp63α was either silenced (sip63) or not silenced (NSC) to optimize the pipeline’s parameters. The small RNA-Seq pipeline optimized and implemented in this study utilizes miRBase v21 as the alignment index and quantitation reference, TMM normalization, and an LNS model for DE analysis. This validated pipeline was used to identify novel ΔNp63α-regulated miRNAs which have predicted roles in cancer signaling. Further characterization of these miRNAs and their mRNA targets may provide mechanistic insight into the progression of non-melanoma skin cancer.

In our analyses, alignments were performed using Bowtie as it is regarded to be “an ultrafast, memory-efficient alignment program for aligning short DNA sequences”^30^. The Johns Hopkins site, which hosts the Bowtie and Bowtie 2 software describes Bowtie as being optimal for sets of short reads where (a) many of the reads have at least one good, valid alignment, (b) many of the reads are relatively high-quality, and (c) the number of alignments per read is small (close to 1)^31^. Accordingly, Bowtie is generally thought to outperform other alignment algorithms for sequences less than 50 bp. While studies such as Ziemann *et al*. have shown that Bowtie 2 outperformed Bowtie in both precision and accuracy, Tam *et al*. has shown that Bowtie and Bowtie2 are similar in accuracy, specificity, and sensitivity as measured by congruence to RT-PCR data (supplemental materials, Tam *et al*.)^20,32^. Bowtie 2 was developed to handle gapped alignments, however, that is not needed for aligning short sequences that span the entire length of the miRNA it resembles.

Using the whole genome and RefSeq indexes for alignment yielded a large number of reads which did not map to known miRNA sequences, suggesting that many reads aligned to non-miRNA loci when these indexes were used (Table 1, Fig. 3). This is likely the result of the presence of other small RNA species that remained after the size exclusion steps of library preparation but may also include a subset of miRNA reads which were misaligned. In either case, these errors would result in artificially low read counts. Quantitation made using the miRBase reference effectively resolved these issues. It is therefore recommended that alignment and quantitation of small RNA-Seq datasets should be performed using the miRBase reference and Bowtie aligner to effectively quantitate unique miRNAs while avoiding the computational time required for aligning to the whole genome or RefSeq indexes. This approach facilitates the rapid quantitation of known miRNAs, allowing researchers to investigate expression level changes and pursue validation experiments. However, it’s worth noting that alignment to the whole genome would be required if the researcher is interested in identifying undiscovered miRNAs.

Previous RNA-Seq pipeline studies have shown that the choice of normalization method can affect the estimation of miRNA abundance and subsequent identification of differentially expressed miRNAs^20,33^. Total count normalization assumes that most RNA are unchanged across samples and scales datasets toward a common distribution, thus TC may be ineffective in situations in which highly expressed miRNAs are differentially expressed^34^. Similarly, the intended utility of TPM and RPKM normalizations in correcting for sequencing biases attributed to read length has been questioned in mRNA-Seq analyses and may actually increase variance in miRNA datasets due to similar biases^35^. All three methods assume similarities between read count distributions and function in a similar manner if corrections for read length bias are not necessary. TMM normalization, by contrast, relies on the assumption that most genes are not differentially expressed, and frequently outperforms other methods when datasets differ in composition^20,35^. Further, TMM is robust for lower coverage data where a high number of genes with zero counts is expected^36^. Since miRNAs are of uniform length, with the majority appearing to be expressed at very low basal levels, TMM normalization would seem appropriate. Our analysis supports the robustness of the TMM method in that it effectively stabilized the median distribution irrespective of the alignment used and identified a panel of miRNAs which were ultimately validated by RT-qPCR (Fig. 5).

Differential expression analysis performed using an LNS response distribution model identified a core set of miRNAs in our sip63 samples which were differentially expressed relative to NSC samples (Fig. 7A). Since sample sizes in NGS experiments are generally low and the read count data is non-normally distributed and continuous in nature, we recommend that LNS should be selected over other response distribution models.

Of the novel p63-regulated miRNAs identified in HaCaT cells and validated in this study (Fig. 8), several have known roles in cancer. It is important to note that differential expression of these miRNAs was validated in HaCaT cells, and p63-regulation may be cell type specific. miR-18a-5p plays an oncogenic role in nasopharyngeal cancer by regulating E-cadherin and K-ras^37^. miR-19b-1-5p is downregulated in CD44 + cervical cancer cells which express increased p63 levels, although no link to p63 was implied^38^. miR-20a-5p targets p63 to regulate p53 and PTEN expression, although the feedback regulation of mir-20a-5p by p63 has not been shown^39^. miR-590-5p attenuates the TGFβ signaling pathway through down-regulation of SMAD3, and may regulate cell proliferation, apoptosis and migration^40^. Lastly, miR-93-5p is elevated in colorectal cancer and is known to target WNK lysine deficient protein kinase 1 (WNK1) to inhibit the invasive potential of triple-negative breast cancer cells^41^. Nothing has been published regarding the functional roles or regulation for the remaining miRNAs identified in this study. It is our hope that these miRNAs and additional targets identified by our small RNA-Seq pipeline will be a source of biomarkers and therapeutic targets for p63-related pathologies and provide critical insight into the role played by p63 in cancer.

The analysis presented herein utilized small RNA libraries which were sequenced on the Ion Torrent platform. Lahens *et al*. demonstrated that Illumina and Ion Torrent platforms in RNA-Seq datasets yielded >80% agreement in differential expression with low read depth likely contributing to differences between the platforms^42^. Since our proposed pipeline filters out low read depth miRNAs (<10 reads per sample), we expect that it would perform similarly using Illumina datasets. However, the Lahens study also reported that the choice of alignment reference yielded some differences that were platform-specific. Thus, the pipeline parameters utilized herein should be empirically tested for other sequencing platforms.

Our investigation of the various alignment and quantitation references (whole genome, RefSeq and miRBase) and normalization methods (TC, RPKM, TPM, and TMM) highlights the potential impact of each on the analysis of small RNA-Seq data. While it is important to experiment with pipeline parameters to accommodate differences in sample library composition and confirm data output by RT-qPCR, we propose that miRBase alignment using Bowtie, quantitation with miRBase, and normalization with TMM as performed in Partek Flow provides a robust pipeline for small RNA-Seq analysis, circumventing the need for command line experience.

## Materials and Methods

### Cell culture, reagents, and plasmids

HaCaT, a non-tumorigenic immortalized human keratinocyte cell line, was obtained from Dr. Nancy Bigley (Wright State University, Dayton, OH). Cells were maintained in DMEM Hyclone media (GE Healthcare Life Sciences, Pittsburg, PA) supplemented with 8% fetal bovine serum, 100 U/mL penicillin, and streptomycin at 37 °C in 5% CO_2_.

### siRNA Knockdown

HaCaT cells were transfected with siRNA against p63 (sip63) or non-silencing control (NSC) (Qiagen, Valencia, CA) using Lipofectamine RNAi-Max (Thermo Fisher Scientific, Carlsbad, CA) as previously described^43^.

### Immunoblot analysis

Whole cell extracts were prepared by washing cells in cold 1% Phosphate-Buffered Saline (PBS) and lysing in phosphatase inhibitor buffer (50 mM Tris-HCl [pH 8.0], 120 mM NaCl, 5 mM EGTA, 1 mM EDTA, 5 mM NaPP, 10 mM NAF, 30 mM PMSF, 0.2 mM PMSF, 1 mM Benzamidine, 0.1% NP-40, 100 nM NaVO_4_) supplemented with protease inhibitor cocktail (catalog #P8340, Sigma-Aldrich, St. Louis, MO). Protein was quantitated by Bicinchoninic Assay (BCA) according to the manufacturer’s instructions (Thermo Fisher Scientific, Fremont, CA). Equivalent amounts of protein were separated on 10% SDS-PAGE and transferred to Polyvinylidene difluoride (PVDF) membrane at 350 mA for 1 hour. ΔNp63α and β-actin were detected using rabbit polyclonal anti-p63 (N2C1, GeneTex, Irvine, CA) or mouse monoclonal anti-β-actin (AC15, Santa Cruz Biotechnology, Dallas, TX) antibodies, respectively. HRP-tagged secondary antibodies (Promega, Madison, WI) were used to enable chemiluminescent detection with the Western Lightning Plus-ECL Chemiluminescent Substrate kit (Perkin Elmer, Waltham, MA).

### Small RNA-Seq library preparation and sequencing

Total RNA was isolated from HaCaT cells using the mirVana^TM^ Paris Kit (Thermo Fisher Scientific, Carlsbad, CA) and enriched for small RNA through size selection ethanol washes. For each library, 4 ng of miRNA was hybridized and ligated to Ion Adapters v2 (Ion Total RNA-Seq Kit v2, Life Technologies, Carlsbad, CA). Small RNA samples were reverse transcribed to cDNA using adapter specific Ion RT primers v2 (Life Technologies, Carlsbad, CA). Purified cDNA samples were size-selected and amplified by PCR followed by further purification and size selection. cDNA samples were barcoded using Platinum PCR SuperMix High Fidelity polymerase with IonXpress RNA 3′ Barcode primer and unique 5′ Ion Xpress RNA-Seq Barcode Primers using the Ion Xpress RNA-Seq Barcode 1–16 Kit (catalog #4471250, Life Technologies, Carlsbad, CA). Yield and size distribution of the cDNA libraries were assessed using the Agilent 2100 Bioanalyzer DNA1000 chip (catalog #5067–1504, Agilent Technologies, Santa Clara, CA). Total barcoded cDNA within the 50–300 base pair range was considered to be derived from small RNA. 7.5 picomoles of each barcoded library were pooled and clonally amplified onto Ion Sphere^TM^ Particles (ISPs) according to the manufacturer’s protocol (Ion PI Template OneTouch^TM^ 200 Template Kit v3) and enriched using the Ion OneTouch 2 ES system (Life Technologies, Carlsbad, CA). Clonal amplification of the cDNA libraries onto ISPs yielded 18.4% templated Ion Sphere Particles (ISPs), well within the manufacturer stated optimal range of 10% to 25% (Ion Sphere^TM^ Quality Control assay, Life Technologies, Carlsbad, CA). Enriched ISPs were sequenced on the Ion Proton Next Generation Sequencing system using the Ion P1 chip v2 Kit and Ion PI ^TM^ Sequencing 200 kit v3 (catalog #4482321 and #4488315, Life Technologies, Carlsbad, CA) with 500 Sequencing flows.

### Small RNA-Seq data analysis

The general workflow for small RNA-Seq analysis used in this study, including alignment, quantitation, normalization, and differential gene expression analysis is shown in Fig. 2. All analyses were performed in Partek® Flow® software, version 5.0 (Copyright 2016, Partek Inc., St. Louis, MO).

### Alignment and Quantification

Sequenced reads were assigned to their respective samples based on corresponding IonXpress barcodes and output in FASTQ format with their associated base quality scores using the Ion Torrent Suite version 4.0.2. FASTQ files were uploaded into Partek Flow software (Partek Inc., St. Louis, MO) for processing. Unaligned reads were trimmed from the 3′ end to a fixed length dependent on the positional base at which the PHRED quality score fell below 20 (Fig. 2A). A minimum read length filter retaining reads greater than 15 bases in length was used in this study and is within the range of 10–26 bases as previously reported^44–47^.

Trimmed reads were aligned to either Whole Genome, RefSeq Transcripts 80 (2-6-2017), or miRBase mature miRNAs version 21 of the latest human genome assembly, hg38 (GRCh38) (Fig. 2B) using the Bowtie aligner^30^. A seed mismatch limit of 1 and minimum seed length of 10 were used. A seed mismatch of 1 was chosen to avoid discarding reads potentially containing inaccurate base calls made by the sequencer. These settings are consistent with the standard recommendation provided by Partek Flow software (verbal communication from Partek Inc., St. Louis, MO). The alignment reporting option was set to 200 alignments per read in order to maximize the predictive power of the Expectation Maximization (EM) algorithm without being overly computationally intensive^48^.

Raw read counts were obtained by quantitating aligned reads to either RefSeq Transcripts 80 (2-6-2017) or miRBase version 21, using a modified version of the EM algorithm implemented by Xing *et al*. in which isoform expression levels are quantified across the whole genome at the same time (Fig. 2C)^49^. Details of the Partek EM algorithm can be found in the White Paper on RNA-Seq Methods^50^. The EM algorithm is used to resolve ambiguous mappings (i.e. reads aligning well to multiple loci) for improved estimation of true expression read counts. It assigns an initial estimate of transcript abundance derived from uniquely mapped reads and then employs a Bayesian approach to calculate the most likely alignment for reads that map to multiple locations on the reference genome. These alignments are used to re-compute global transcript abundances, which are utilized to probabilistically re-align and resolve ambiguous mappings. This process is iterated until the algorithm converges, at which point the reads assigned to a particular locus are counted, resulting in final raw read counts.

### Normalization

Raw read counts were normalized using Total Count (TC), Reads Per Kilobase per Million (RPKM), Transcripts Per Million (TPM), or Trimmed Mean of M values (TMM) normalization methods^34–36,51,52^ (Fig. 2D). Since miRNAs with 0 read counts would impede statistical calculations when performing differential expression analysis, an offset of 1.0 was added to all normalized read counts. This facilitated reporting for all annotated miRNAs at this stage. The offset did not result in inclusion of miRNAs with near zero read counts in the final DE lists since these lists were filtered to include only miRNAs with minimum read count values of greater than or equal to 10 reads in each of the profiled samples.

### Differential Expression (DE) Analysis

Normalized read counts for each miRNA were statistically modeled using Partek Flow’s Gene Specific Analysis (GSA) approach. The GSA approach uses the data to select the best model (Normal (N), Negative Binomial (NB), Lognormal (LN), and Lognormal with Shrinkage (LNS)) for each miRNA based on the lowest corrected Akaike Information Criterion corrected (AICc)^53^. Because the LNS model yielded the lowest AICc for a majority (72%) of miRNAs identified, it was selected as the default model for DE analysis. A low expression filter based on Lowest Average Coverage (LAC) was set with a cutoff of 4, thereby excluding miRNAs with a geometric mean across all samples below this value (Fig. 2E). LNS utilizes an empirical Bayes method that estimates gene-specific dispersion by combining information about variance from other genes to improve the estimation process, resulting in improved DE detection and lower false-positive rates^54^. The LNS model is similar to the “limma trend” method from the limma package previously reported to be a robust model for differential expression analysis and is generally beneficial when the number of experimental replicates is low^46,55^. P-values were generated using the F statistic. P-values less than or equal to 0.05 were deemed significant.

### Reverse Transcription Quantitative Polymerase Chain Reaction (RT-qPCR)

Total RNA was extracted from HaCaT cells transfected with siRNA against p63 (sip63) and non-silencing control (NSC) using the mirVana^TM^ Paris^TM^ Isolation kit according to the manufacturer’s protocol (Thermo Fisher Scientific, Carlsbad, CA). 1 µg of total RNA input was used for cDNA synthesis using the q-Script cDNA supermix (Quanta Biosciences, Beverly, MA). TaqMan based RT-qPCR was performed as previously described^43^. Assays on Demand (AODs) for p63 (Hs00978340_ml) and GAPDH (4325792) (Thermo Fisher Scientific, Carlsbad, CA, USA) were used. p63 expression was normalized to GAPDH according to the manufacturer’s instructions (Thermo Fisher Scientific, Carlsbad, CA). For miRNA quantitation, 10 ng of RNA was used for cDNA synthesis using the TaqMan miRNA reverse transcription kit according to manufacturer’s instructions (Applied Biosystems, Japan Ltd). qPCR was performed using the Universal TaqMan master mix (2X) and miRNA-specific TaqMan AODs for hsa-miR-149-5p (002255), hsa-miR-18a-5p (002422), hsa-miR-19b-1-5p (002425), hsa-miR-20a-5p (000580), hsa-miR590-5p (001984), hsa-miR-744-5p (002324), hsa-miR-93-5p (001090) and RNU48 (001006) (Thermo Fisher Scientific, Carlsbad, CA). MiRNA expression was normalized to RNU48 according to the manufacturer’s instructions (Thermo Fisher Scientific, Carlsbad, CA). Student’s t-tests were used to determine significant differences in sip63 samples relative to NSC controls.

### Ingenuity Pathway Analysis (IPA) of DE miRNA

The functional roles and signaling networks of p63-regulated miRNA were identified using IPA (Qiagen Inc., Valencia, CA, https://www.qiagenbioinformatics.com/products/ingenuitypathway-analysis). The algorithms developed for use in IPA have been previously described^56^. The input dataset for this analysis was the list of 79 significant DE miRNAs obtained from alignment and quantitation to the miRBase index and normalization by TMM after filtering on p ≤ 0.05, fold-change ≥1.5 and read counts ≥10 in all samples. MiRNAs were associated with their respective experimentally validated mRNA targets by querying the Ingenuity Pathways Knowledge Base (Qiagen Inc., Valencia, CA). Functional profiling was performed to identify cellular processes which showed enrichment for these mRNA. The upstream analysis function in IPA was used to filter the list of experimentally validated mRNA targets to only those with known links to p63 (TP63) in the IPA Knowledge Base. The IPA network connection tools were then used to display known functional connections between differentially expressed miRNA and these mRNA targets. Signaling network generation was performed using the Path Designer tools in IPA (Qiagen, Inc., Valencia, CA).

### Statistical analysis

Relative log expression (RLE) plots were generated according to established methods^57^. Briefly, 1) read counts were log_10_ transformed, 2) the median of log expression values for each miRNA across all samples was calculated, 3) RLE values were calculated by subtracting this median value from each of the miRNA log read count value for each sample to obtain an RLE matrix, and 4) a box plot of RLE values was generated for each NSC and sip63 sample.

Principal components analysis (PCA) was performed to assess the overall variability in the miRNA datasets. The statistical significance of the separation of sip63 and NSC samples in PCA space was determined by a permutation test of the Davies-Bouldin (DB) index measure run with 1,000 iterations^58^. DB index compares the intra-cluster distances among samples to the distance between cluster centroids; smaller values indicates a better separation of samples belonging to different groups. Statistical significance of group separation in PCA space was assessed by permutation analysis of Davies-Bouldin index as described^59^.

Hierarchical cluster analysis (HCA) was performed on the subset of miRNA genes that satisfied differential expression criteria. The dataset was normalized by the root-mean-square algorithm applied across genes, and experiments were median-centered. HCA was run with Euclidean distance measure and average linkage clustering option.

### Data Availability

The datasets generated during and/or analyzed during the current study are available from the corresponding author on reasonable request.

## Electronic supplementary material

Dataset 1 and Dataset 2
