# Supplementary material for: Identification of novel ΔNp63α-regulated miRNAs using an optimized small RNA-Seq analysis pipeline
Source: Sci Rep. 2018 Jul 3;8:10069. doi: 10.1038/s41598-018-28168-5 (PMC6030203; doi:10.1038/s41598-018-28168-5)
Supplement: Supplementary file 1 — Dataset 1 and Dataset 2 [file 41598_2018_28168_MOESM1_ESM.docx]

# Identification of novel ΔNp63α-regulated miRNA using an optimized small RNA-Seq analysis pipeline

Suraj Sakaram1, Michael P. Craig1, Natasha Hill1 Amjad Aljagthmi1, Christian Garrido1, Oleg Paliy1, Michael Bottomley2, Michael Raymer3 and Madhavi Kadakia1*

1 Biochemistry and Molecular Biology, Wright State University, Dayton OH 45435, USA

2 Math and Microbiology, Wright State University, Dayton OH 45435, USA

3 Computer Science and Engineering, Wright State University, Dayton OH 45435, USA

* To whom correspondence should be addressed. Tel: 937-775-2339; Fax: (937) 775-3730; Email: [Madhavi.Kadakia@wright.edu](mailto:Madhavi.Kadakia@wright.edu)

# Supplementary Information

**Supplementary Table S1:** miRNA positively regulated by ΔNp63α which were identified as differentially expressed by aligning and quantifying to miRBase, normalizing by TMM using the LNS method. miRNA listed are those with reads ≥ 10 for all samples, fold-change (FC) ≤ -1.5 with p ≤ 0.05. Shown are the average read counts ± 1 SD for NSC and sip63 samples (n = 3 each), p-value calculated in Partek Flow using the LNS fit model, and fold-change (FC).

| **microRNA ID** | **NSC average reads** | **sip63 average reads** | **p** | **FC** |
| --- | --- | --- | --- | --- |
| let-7f-1-3p | 456 ± 59 | 250 ± 50 | 0.02 | -1.82 |
| let-7f-2-3p | 644 ± 116 | 389 ± 17 | 0.04 | -1.66 |
| miR-100-5p | 7,676 ± 1095 | 4,254 ± 957 | 0.02 | -1.80 |
| miR-107 | 7,531 ± 1347 | 4,022 ± 1,088 | 0.01 | -1.87 |
| miR-1275 | 319 ± 59 | 178 ± 2 | 0.02 | -1.79 |
| miR-1306-5p | 50 ± 10 | 24 ± 10 | 0.02 | -2.06 |
| miR-1307-5p | 643 ± 386 | 252 ± 108 | 0.03 | -2.55 |
| miR-130a-3p | 2,335 ± 518 | 1,246 ± 190 | 0.01 | -1.87 |
| miR-130b-3p | 1,334 ± 124 | 781 ± 185 | 0.03 | -1.71 |
| miR-135b-5p | 179 ± 73 | 75 ± 40 | 0.01 | -2.40 |
| miR-149-5p | 1,166 ± 67 | 704 ± 67 | 0.03 | -1.66 |
| miR-15a-3p | 53 ± 16 | 26 ± 6 | 0.03 | -2.01 |
| miR-181d-5p | 391 ± 115 | 185 ± 80 | 0.02 | -2.11 |
| miR-185-5p | 2,965 ± 286 | 1,648 ± 343 | 0.02 | -1.80 |
| miR-18a-5p | 7,836 ± 922 | 4,131 ± 854 | 0.01 | -1.90 |
| miR-18b-5p | 7,462 ± 884 | 3,377 ± 1,006 | <0.01 | -2.21 |
| miR-19b-1-5p | 332 ± 48 | 170 ± 51 | 0.01 | -1.95 |
| miR-203a-5p | 98 ± 7 | 54 ± 13 | 0.03 | -1.80 |
| miR-205-3p | 472 ± 109 | 237 ± 76 | 0.01 | -1.99 |
| miR-205-5p | 282,536 ± 29,502 | 104,606 ± 29,228 | <0.01 | -2.70 |
| miR-20a-5p | 46,862 ± 10,526 | 24,879 ± 3,566 | 0.01 | -1.88 |
| miR-210-3p | 1,910 ± 367 | 1,001 ± 217 | 0.01 | -1.91 |
| miR-211-5p | 38 ± 3 | 20 ± 5 | 0.03 | -1.93 |
| miR-215-5p | 479 ± 59 | 286 ± 75 | 0.04 | -1.67 |
| miR-27a-5p | 310 ± 16 | 171 ± 63 | 0.02 | -1.82 |
| miR-29b-1-5p | 512 ± 230 | 278 ± 139 | 0.05 | -1.85 |
| miR-3124-3p | 87 ± 22 | 44 ± 4 | 0.02 | -1.95 |
| miR-3141 | 123 ± 20 | 71 ± 24 | 0.04 | -1.75 |
| miR-3529-3p | 16,424 ± 3,476 | 7,221 ± 818 | <0.01 | -2.27 |
| miR-363-5p | 65 ± 21 | 30 ± 5 | 0.02 | -2.18 |
| miR-378a-3p | 2,770 ± 1,149 | 1,024 ± 413 | 0.01 | -2.70 |
| miR-378a-5p | 360 ± 135 | 176 ± 58 | 0.02 | -2.05 |
| miR-421 | 252 ± 60 | 108 ± 24 | <0.01 | -2.33 |
| miR-423-3p | 1,924 ± 40 | 1,024 ± 258 | 0.01 | -1.88 |
| miR-424-5p | 616 ± 76 | 356 ± 6 | 0.02 | -1.73 |

**Supplementary Table S1** (continued)

| **microRNA ID** | **NSC average reads** | **sip63 average reads** | **p** | **FC** |
| --- | --- | --- | --- | --- |
| miR-429 | 3,172 ± 702 | 1,898 ± 328 | 0.04 | -1.67 |
| miR-455-3p | 789 ± 218 | 368 ± 221 | 0.01 | -2.15 |
| miR-4647 | 32 ± 1 | 18 ± 2 | 0.04 | -1.81 |
| miR-4677-5p | 50 ± 11 | 27 ± 11 | 0.04 | -1.86 |
| miR-503-5p | 501 ± 30 | 280 ± 65 | 0.02 | -1.79 |
| miR-513b-3p | 25 ± 5 | 13 ± 1 | 0.04 | -1.91 |
| miR-548t-3p | 110 ± 15 | 65 ± 6 | 0.04 | -1.70 |
| miR-590-3p | 289 ± 166 | 85 ± 32 | 0.01 | -3.42 |
| miR-590-5p | 1,425 ± 407 | 816 ± 136 | 0.04 | -1.75 |
| miR-615-3p | 347 ± 36 | 182 ± 11 | 0.01 | -1.91 |
| miR-660-5p | 190 ± 18 | 112 ± 19 | 0.04 | -1.70 |
| miR-671-5p | 376 ± 102 | 178 ± 53 | 0.01 | -2.11 |
| miR-6807-5p | 31 ± 7 | 16 ± 2 | 0.04 | -1.90 |
| miR-744-5p | 753 ± 85 | 391 ± 66 | 0.01 | -1.93 |
| miR-7974 | 74 ± 15 | 39 ± 4 | 0.02 | -1.90 |
| miR-7975 | 3,954 ± 588 | 1,888 ± 540 | 0.01 | -2.09 |
| miR-877-3p | 182 ± 28 | 103 ± 27 | 0.03 | -1.77 |
| miR-924 | 114 ± 69 | 38 ± 32 | 0.02 | -3.05 |
| miR-92a-1-5p | 68 ± 23 | 29 ± 14 | 0.01 | -2.31 |
| miR-93-5p | 15,979 ± 4,754 | 8,320 ± 2,402 | 0.02 | -1.92 |
| miR-942-3p | 47 ± 3 | 27 ± 7 | 0.04 | -1.78 |
| miR-99a-5p | 6,747 ± 854 | 3,451 ± 794 | 0.01 | -1.96 |
| miR-99b-5p | 1,825 ± 562 | 1,011 ± 322 | 0.03 | -1.80 |

**Supplementary Table S2:** miRNA negatively regulated by ΔNp63α which were identified as differentially expressed by aligning and quantifying to miRBase, normalizing by TMM using the LNS method. miRNA listed are those with reads ≥ 10 for all samples, fold-change (FC) ≥ 1.5 and p ≤ 0.05. Shown are the average read counts ± 1 SD for NSC and sip63 samples (n = 3 each), p-value calculated in Partek Flow using the LNS fit model, and FC.

| **microRNA ID** | **NSC average reads** | **sip63 average reads** | **p** | **FC** |
| --- | --- | --- | --- | --- |
| miR-10a-3p | 510 ± 205 | 909 ± 291 | 0.05 | 1.78 |
| miR-1181 | 384 ± 73 | 640 ± 125 | 0.04 | 1.67 |
| miR-3138 | 16 ± 3 | 34 ± 8 | 0.02 | 2.15 |
| miR-3912-3p | 20 ± 3 | 36 ± 5 | 0.04 | 1.85 |
| miR-3925-3p | 16 ± 1 | 30 ± 2 | 0.03 | 1.90 |
| miR-3976 | 70 ± 9 | 135 ± 54 | 0.05 | 1.91 |
| miR-4308 | 20 ± 8 | 61 ± 10 | <0.01 | 3.09 |
| miR-4515 | 48 ± 3 | 99 ± 35 | 0.02 | 2.06 |
| miR-4750-3p | 344 ± 107 | 648 ± 212 | 0.03 | 1.88 |
| miR-5697 | 121 ± 16 | 209 ± 10 | 0.03 | 1.73 |
| miR-582-3p | 154 ± 52 | 357 ± 89 | <0.01 | 2.31 |
| miR-628-5p | 18 ± 3 | 35 ± 10 | 0.04 | 1.95 |
| miR-6749-3p | 15 ± 3 | 34 ± 5 | 0.01 | 2.25 |
| miR-6760-5p | 34 ± 3 | 71 ± 17 | 0.01 | 2.06 |
| miR-6817-5p | 20 ± 9 | 37 ± 5 | 0.04 | 1.84 |
| miR-6850-5p | 42 ± 2 | 149 ± 57 | <0.01 | 3.57 |
| miR-6854-3p | 16 ± 3 | 29 ± 2 | 0.04 | 1.84 |
| miR-6864-5p | 18 ± 3 | 42 ± 16 | 0.02 | 2.30 |
| miR-8065 | 49 ± 7 | 85 ± 18 | 0.05 | 1.73 |
| miR-887-3p | 28 ± 13 | 62 ± 31 | 0.03 | 2.22 |
| miR-938 | 15 ± 4 | 29 ± 9 | 0.05 | 1.95 |
